# A Case of Acute Pericarditis and Hypereosinophilia After Dupilumab Initiation

**DOI:** 10.7759/cureus.105073

**Published:** 2026-03-11

**Authors:** Tiffany Truong, Ani Shirvanian, Marcel Hariri, Roy Artal, Sherwin Hariri

**Affiliations:** 1 Department of Medicine, Cedars-Sinai Medical Center, Los Angeles, USA; 2 Department of Allergy and Immunology, Cedars-Sinai Medical Center, Los Angeles, USA; 3 Department of Allergy and Immunology, Rose and Alex Pilibos Armenian School, Los Angeles, USA; 4 Division of Pulmonary and Critical Care Medicine, Department of Medicine, Cedars-Sinai Medical Center, Los Angeles, USA

**Keywords:** acute pericarditis, acute pericarditis after dupilumab, dupilumab, eosinophilic pericarditis, hypersensitivity pericarditis

## Abstract

Dupilumab is a monoclonal antibody approved to treat multiple type 2 inflammatory conditions. Dupilumab-associated hypereosinophilia is a well-documented side effect and typically does not lead to clinical symptoms or pathologic findings. We report a 65-year-old man with asthma and nasal polyposis who presented with pleuritic chest pain after two doses of dupilumab. Our evaluation revealed peripheral hypereosinophilia and pericardial thickening consistent with pericarditis on cardiac MRI. The patient was treated with ibuprofen and colchicine, did not require corticosteroids, and dupilumab was discontinued. Subsequently, his eosinophil counts normalized, and his symptoms resolved. To our knowledge, this is the first published case of dupilumab-induced hypereosinophilia followed by isolated pericardial involvement.

## Introduction

Dupilumab is a monoclonal antibody that targets the interleukin-4 (IL-4) receptor alpha subunit, thereby inhibiting IL-4 and IL-13 signaling [1]. Dupilumab is FDA-approved for multiple type 2 inflammatory conditions, including asthma, atopic dermatitis, eosinophilic esophagitis, chronic rhinosinusitis with nasal polyps, and prurigo nodularis [2]. Despite its favorable safety profile, dupilumab has been associated with peripheral eosinophilia, a well-described laboratory finding that is typically transient and does not present with clinical symptoms [3]. The proposed mechanism of eosinophilia involves IL-4 and IL-13 inhibition, resulting in downregulation of chemokines and adhesion molecules involved in eosinophil tissue migration, therefore increasing circulating eosinophils [1]. Rare cases of clinically significant eosinophilic complications have been reported for patients on dupilumab, including eosinophilic granulomatosis with polyangiitis (EGPA) and eosinophilic pneumonia [3, 4].

To our knowledge, this is the first published case describing a patient with hypereosinophilia and isolated acute pericarditis after starting dupilumab.

## Case presentation

A 65-year-old man with a medical history of hypertension, hyperlipidemia, non-obstructive coronary artery disease, sleep apnea, asthma, and nasal polyposis presented to the emergency department with two months of pleuritic, substernal chest pain. He had started dupilumab therapy for nasal polyposis and persistent nasal congestion. His symptoms began shortly after the second dose and persisted despite completion of a total of five doses by the time of presentation. His chest pain was non-radiating and accompanied by exertional dyspnea, palpitations, and lightheadedness. He denied fever, chills, recent travel, rashes, myalgias, or joint pain.

The patient's home medications included a combination inhaler of fluticasone furoate, umeclidinium, and vilanterol; monthly allergen immunotherapy; and dupilumab 300 mg biweekly. He reported no family history of similar presentations and had no known drug allergies.

**Figure 1:**
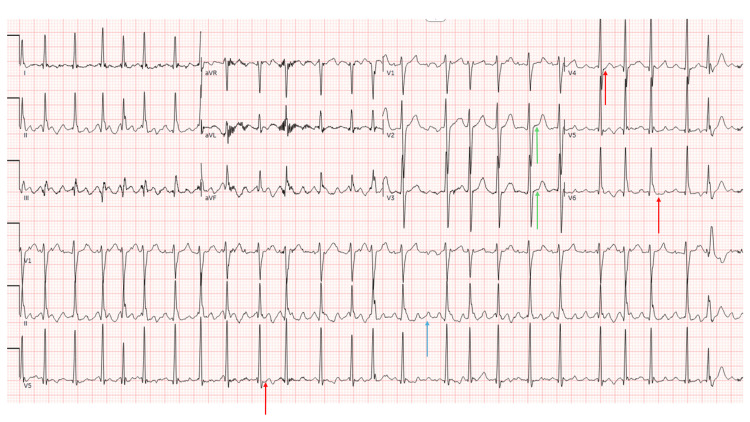
Electrocardiogram (EKG) EKG in the emergency room showing atrial flutter with a rapid ventricular rate of 150. The blue arrow denotes the saw-tooth pattern seen in atrial flutter. Red arrows indicate mild rate-related ST depressions. Green arrows highlight mild, non-specific ST elevations in leads V2 and V3, rather than diffuse ST elevations classically associated with acute pericarditis.

Vital signs on presentation were notable for tachycardia in the 150s due to new-onset atrial flutter with a rapid ventricular rate, which spontaneously converted to normal sinus rhythm shortly thereafter and did not recur on telemetry while inpatient. An electrocardiogram (EKG) was obtained on arrival in the emergency room (Figure 1).

**Figure 2:**
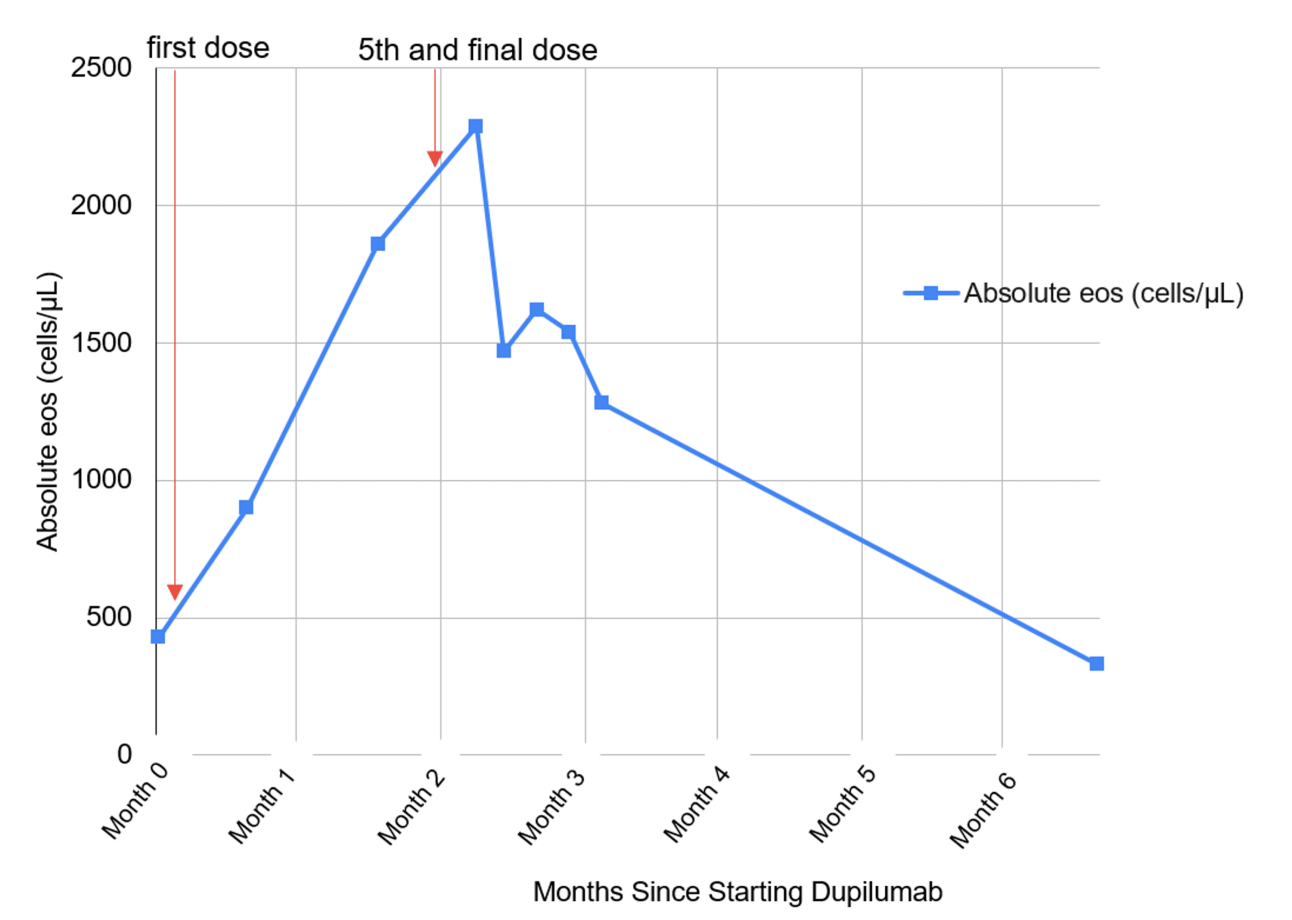
Peripheral Eosinophil Trend Peripheral eosinophil counts before dupilumab initiation and after its cessation. Red arrows show the first and final doses of dupilumab. eos: esonophil.

Physical examination was unremarkable. Laboratory tests revealed an elevated C-reactive protein of 20 mg/L (reference range: <8 mg/L) and an erythrocyte sedimentation rate of 51 mm/hr (reference range: <20 mm/hr). His absolute eosinophil count peaked at 2,300 cells/μL (reference range: <400 cells/μL), with a sharp increase in eosinophil counts after starting dupilumab and normalizing eosinophil counts after cessation of the medication (Figure 2).

A rheumatologic workup was performed and was unremarkable apart from a weakly positive anti-nuclear antibody. Anti-neutrophil cytoplasmic antibodies (ANCA), anti-Jo-1 antibody, creatine kinase, anti-double-stranded DNA antibody, anti-ribosomal antibody, anti-Smith antibody, anti-SS-A antibody, anti-SS-B antibody, anti-Scl-70 antibody, anti-centromere antibody, anti-RNA polymerase III antibody, anti-cyclic citrullinated peptide antibody, and rheumatoid factor were negative.

The remainder of his complete blood count, metabolic panel, troponin, urinalysis, and thyroid function tests were within normal limits. His infectious workup was negative and included an acute hepatitis panel, human immunodeficiency virus (HIV) antigen/antibody, QuantiFERON-TB Gold, an extended respiratory viral panel, and a Strongyloides antibody.

**Figure 3:**
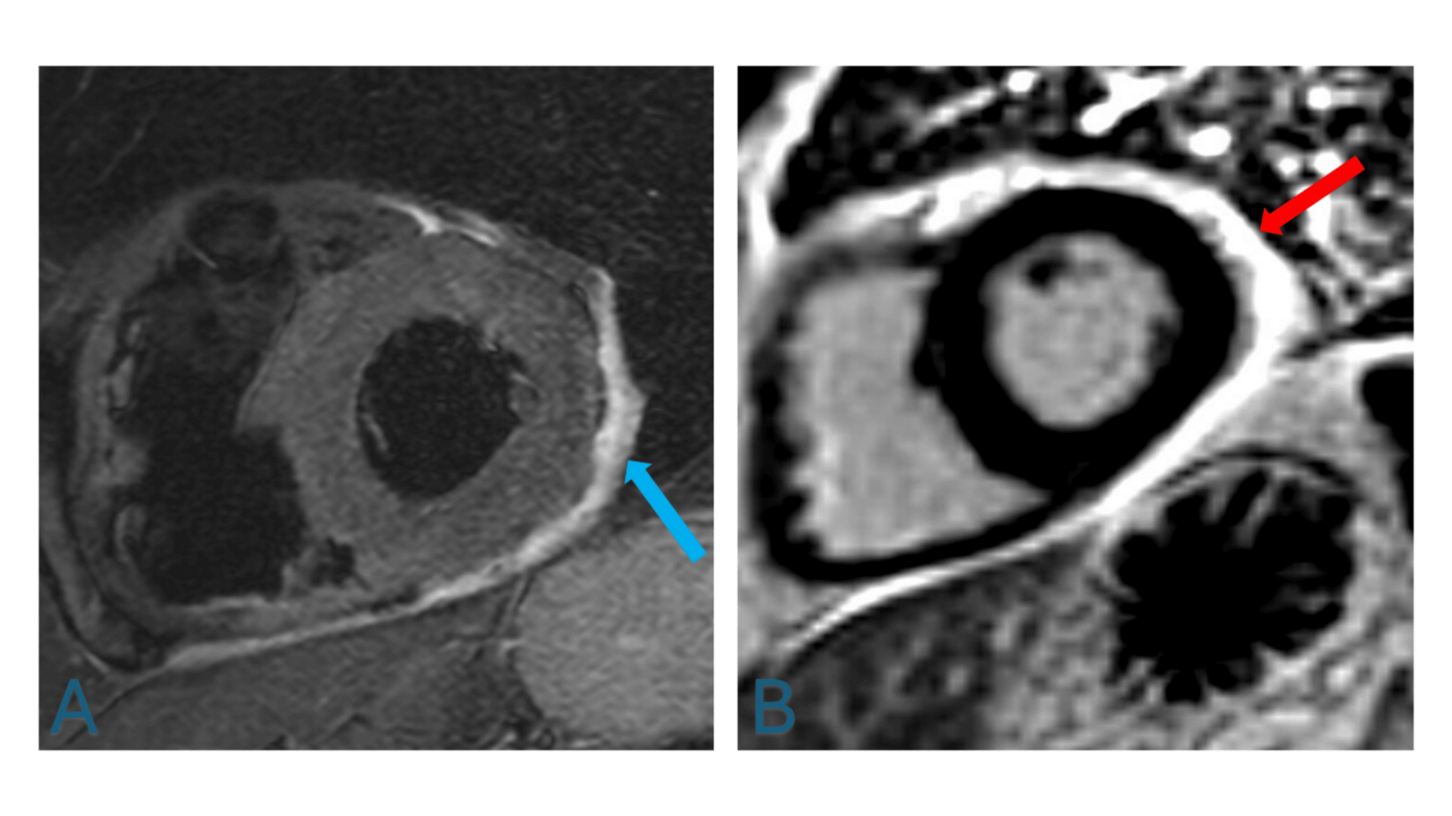
Cardiac Magnetic Resonance Imaging Short axis short tau inversion recovery (STIR) fluid-sensitive (A) and delayed post-gadolinium contrast cardiac MRI sequences (B). Note the bright, hyperintense signal on the STIR sequence indicating pericardial edema (blue arrow) and late post-gadolinium contrast enhancement involving the thickened pericardium (red arrow), consistent with pericarditis and associated pericardial effusion.

A computed tomography (CT) of his sinuses showed only bilateral sinonasal polyps. A chest CT showed no change in his chronic bilateral small pleural effusions with associated atelectasis. Transthoracic echocardiography demonstrated normal systolic and diastolic function, pulmonary artery pressure, valvular function, pericardium without pericardial effusion, and a lack of regional wall motion abnormalities. However, cardiac magnetic resonance imaging (MRI) revealed a markedly thickened pericardium with prominent pericardial edema, trace effusion, and absence of myocardial edema (Figure 3).

He was admitted for three days and diagnosed with acute pericarditis. Dupilumab was discontinued, and his symptoms improved with colchicine 0.6 mg twice daily and ibuprofen 600 mg three times daily. Steroids were considered but deemed unnecessary given his clinical improvement on colchicine and ibuprofen alone. Given the onset of symptoms and peripheral eosinophilia after the initiation of dupilumab, along with subsequent clinical improvement after its cessation, eosinophilic pericarditis was strongly suspected. Pericardial biopsy was not considered necessary for diagnosis due to the high level of clinical confidence, rapid symptom improvement on therapy, and because the potential risks and patient discomfort were felt to outweigh the expected diagnostic benefit. At outpatient follow-up one week later, his inflammatory markers had decreased, absolute eosinophil count had normalized, and his pleuritic chest pain and dyspnea had almost completely resolved.

## Discussion

In this case, our patient developed a rare and severe complication of hypereosinophilia with associated pericarditis after receiving two dupilumab doses. We believe the patient experienced a hypersensitivity reaction to dupilumab, manifesting as eosinophilic pericarditis. The Naranjo Adverse Drug Reaction Probability Scale is a widely used standardized tool to assess the likelihood of causality for adverse drug reactions [5]. Application of this scale yielded a score of 6 out of 9, corresponding to a “probable” adverse drug reaction, based on the temporal relationship between symptom onset and drug administration, the presence of objective evidence of an adverse reaction, and the absence of a reasonable alternative explanation.

Infectious etiologies of pericarditis were considered unlikely given the absence of clinical signs of infection, no recent illness or relevant exposures, and a negative viral workup. Autoimmune causes were similarly deemed less likely due to the absence of joint or systemic symptoms and negative rheumatologic laboratory evaluation. Metabolic etiologies were excluded based on a normal comprehensive metabolic panel and thyroid function testing. There was no clinical history to suggest a traumatic etiology, and normal cardiac biomarkers and lack of wall motion abnormalities on cardiac imaging did not support an ischemic cause.

In rare cases, EGPA has been reported following dupilumab use [6]. EGPA was considered, given his history of asthma and chronic rhinosinusitis with nasal polyposis. However, the diagnosis was deemed less likely due to the absence of neurological symptoms, negative ANCA and rheumatologic workup, and a lack of vasculitic findings. Additionally, there was a clear alternative explanation for his transient hypereosinophilia, supported by his clinical improvement following drug cessation.

His new-onset atrial flutter was attributed to acute illness and inflammation from acute pericarditis, and myocarditis was excluded based on cardiac MRI findings without myocardial edema. Additionally, signs of constrictive pericarditis, such as diastolic septal flattening, atrial dilation, and dilated vena cava, were not seen on imaging. Our patient’s presentation with acute pericarditis is clinically distinct from acute myocarditis and represents the first reported case in the literature to be associated with dupilumab initiation.

Naidu and Vatti (2025) reported a similar case in which a patient developed hypereosinophilia-related myocarditis following the initiation of dupilumab therapy [7]. Resolution of symptoms was achieved with high-dose corticosteroids and discontinuation of dupilumab. A systematic review article in 2017 identified that the majority of reported cases of eosinophilic myocarditis were treated with corticosteroids, especially if the underlying cause was secondary to hypereosinophilic syndrome or EGPA, and less frequently in hypersensitivity myocarditis [8].

Transient hypereosinophilia is a known and relatively common adverse effect of dupilumab. This occurs through IL-4/IL-13 blockade, which inhibits eosinophil migration into tissues and results in elevated peripheral eosinophil counts [9]. Although hypereosinophilia, secondary to dupilumab, is typically mild, transient, and asymptomatic, it rarely results in clinically significant adverse events [10]. Given the frequency of peripheral eosinophilia during dupilumab therapy and the rarity of related complications, an important clinical question is identifying risk factors that may predispose certain patients to eosinophil-mediated adverse outcomes [11, 12]. In a 2022 analysis of 11 dupilumab clinical trials, Weschler et al. found that among patients with eosinophil counts exceeding 3,000 cells/μL, only seven out of 4,666 participants developed symptoms related to eosinophil-associated adverse effects [3].

Another important consideration is the potential relationship between the degree of eosinophilia and the likelihood of eosinophil-mediated complications; however, current evidence remains insufficient to clearly define this relationship [11]. It is difficult to predict the likelihood of end-organ damage based solely on the number of circulating eosinophils; tissue recruitment, rather than circulating counts, is the more important determinant of damage [13]. A large, multicenter retrospective analysis revealed that peripheral eosinophilia was present in only 57% of 156 histologically confirmed cases of eosinophilic myocarditis, with a median count of 630 cells/μL [14]. Hence, more research is warranted to understand the relationship between dupilumab exposure, eosinophilic pericarditis, and the degree of peripheral eosinophilia.

Given these uncertainties, we recommend that clinicians obtain baseline eosinophil levels prior to initiating dupilumab, followed by close monitoring of eosinophil counts and symptoms of end-organ involvement. Hypereosinophilia, along with chest pain and dyspnea, should alert physicians to the possibility of eosinophilic pericarditis and prompt urgent evaluation. Early recognition of this condition as a rare but potential complication of dupilumab is crucial to avoid delays in diagnosis and management.

## Conclusions

Dupilumab-induced hypereosinophilia is a well-recognized adverse effect that is typically mild and transient in nature. However, it can lead to serious eosinophil-mediated complications in rare cases. Our case represents the first reported instance of dupilumab-induced hypereosinophilia followed by acute pericarditis. Clinicians should monitor for signs of end-organ involvement in patients presenting with elevated eosinophil counts during dupilumab therapy. Early recognition and prompt discontinuation of the drug can lead to symptom resolution, reduce the risk and severity of complications, and prevent recurrence.
